# Exceso de mortalidad invernal en Argentina entre 1997 y 2017: abordaje desde la salud colectiva y disputa epistémica desde el Sur Global

**DOI:** 10.1590/0102-311XES168524

**Published:** 2025-06-20

**Authors:** Virna Almeida, Marcio Alazraqui

**Affiliations:** 1 Departamento de Epidemiología e Información Estratégica en Salud, Hospital de Alta Complejidad El Calafate, El Calafate, Argentina.; 2 Instituto de Salud Colectiva, Universidad Nacional de Lanús, Buenos Aires, Argentina.

**Keywords:** Estudios de Series Temporales, Mortalidad Excesiva, Desigualdad en la Salud, Time Series Studies, Excess Mortality, Health Status Disparities, Estudos de Temporais, Excesso de Mortalidade, Desigualdades em Saúde

## Abstract

El exceso de mortalidad invernal es estudiado en el Norte Global como expresión de desigualdades sociales y mortalidad prevenible. Su determinación es compleja y difiere de la mortalidad general. Nuestro objetivo fue evidenciar la ocurrencia de exceso de mortalidad invernal en Argentina entre 1997 y 2017, describir su magnitud, tendencia y distribución para discutirlo desde una perspectiva crítica como objeto de conocimiento para la salud colectiva. Utilizamos bases de datos de mortalidad oficiales para un estudio de series temporales a nivel país, regiones y provincias, integrando agregados espaciales y temporales. Identificamos 407.950 muertes invernales en exceso, con un promedio anual de 16.667 (20,4%; IC95%: 18,6; 22,2). Este fue mayor en mujeres (21,8%; IC95%: 20,5% 24,9) que en varones (18,2%; IC95%: 16,2; 19,5). El 92,3% afectó a mayores de 60 años, con predominancia en mujeres mayores de 80 años (63%) y varones de 60 a 79 años (49,7%). También se observó exceso de mortalidad invernal en menores de 5 años, pero no en varones de 15 a 29 años. El nadir ocurrió en 2010 (15%; IC95: 14,7%; 15,2) y el pico en 1999 (30,5%; IC95%: 30,2; 30,9). La latitud explicó el 75% de la variabilidad entre provincias (R² ajustado = 0,75). Concluimos que mujeres, menores de 5 años, mayores de 60 años y residentes de regiones Centro y Cuyo fueron los más afectados. Este trabajo visibiliza, a través de un indicador *proxy*, desigualdades en la mortalidad probablemente injustas y reducibles, sintetizando vulnerabilidades individuales y colectivas. Es pionero a nuestro entender, en abordarlo críticamente desde el Sur Global, aportando conocimiento relevante para la salud colectiva.

## Abstract (en)

Excess mortality in winter is studied in the Global North as a manifestation of social inequalities and avoidable deaths. It has a complex identification that differs from general mortality. This study aims to highlight the occurrence of excess winter mortality in Argentina from 1997 to 2017. Also, it describes excess mortality magnitude, trend, and distribution in order to discuss it based on a critical perspective as an object of knowledge for public health. Official mortality databases were used for a study of time series at the national, regional and provincial levels, integrating spatial and temporal aggregates. In total, 407,950 excess deaths were identified in winter, with an annual average of 16,667 (20.4%; 95%CI: 18.6; 22.2). A higher prevalence was found in women (21.8%; 95%CI: 20.5; 24.9) than in men (18.2%; 95%CI: 16.2; 19.5). Of this total, 92.3% occurred in older women aged over 60 years, with a predominance of those older than 80 years (63%) and men aged from 60 to 79 years (49.7%). Excess winter mortality was also found in children aged under 5 years, but not in men aged 15 to 29 years. The nadir occurred in 2010 (15%; 95%CI: 14.7; 15.2) and the peak in 1999 (30.5%; 95%CI: 30.2; 30.9). Latitude explained 75% of the variability between provinces (adjusted R² = 0.75). It is concluded that women, children aged under 5 years, older people aged over 60 years and residents of the Central and Cuyo regions were the most affected. Based on a proxy indicator, this work shows inequalities in mortality that are probably unfair and avoidable, synthesizing individual and collective vulnerabilities. Its pioneering aspect lies in carrying out a critical approach from the Global South, providing knowledge relevant to Public Health.

## Resumo

O excesso de mortalidade invernal é estudado no Norte Global como manifestação de desigualdades sociais e de mortalidade evitável. Tem uma identificação complexa que difere da mortalidade geral. O objetivo deste estudo foi evidenciar a ocorrência do excesso de mortalidade invernal na Argentina entre 1997 e 2017, descrever sua magnitude, tendência e distribuição a fim de discuti-la a partir de uma perspectiva crítica como objeto de conhecimento para a saúde coletiva. Foram utilizadas bases de dados oficiais sobre mortalidade para um estudo de séries temporais em âmbito nacional, regional e provincial, integrando agregados espaciais e temporais. Foram identificadas 407.950 mortes em excesso invernal, com média anual de 16.667 (20,4%; IC95%: 18,6; 22,2). Encontrou-se uma maior prevalência em mulheres (21,8%; IC95%: 20,5; 24,9) do que em homens (18,2%; IC95%: 16,2; 19,5). Desse total, 92,3% ocorreu em idosos acima de 60 anos, com predominância de idosas acima de 80 anos (63%) e homens entre 60 e 79 anos (49,7%). O excesso de mortalidade invernal também foi encontrado em crianças menores de 5 anos, mas não em homens de 15 a 29 anos. O nadir ocorreu em 2010 (15%; IC95%: 14,7; 15,2) e o pico em 1999 (30,5%; IC95%: 30,2; 30,9). A latitude explicou 75% da variabilidade entre as províncias (R² ajustado = 0,75). Conclui-se que as mulheres, crianças menores de 5 anos, idosos acima de 60 anos e moradores das regiões Central e Cuyo foram os mais afetados. A partir de um indicador *proxy*, este trabalho mostra as desigualdades na mortalidade que provavelmente são injustas e evitáveis, sintetizando vulnerabilidades individuais e coletivas. Seu pioneirismo reside em realizar uma abordagem crítica a partir do Sul Global, fornecendo conhecimentos relevantes para a saúde coletiva.

## Introducción

El exceso de mortalidad invernal es objeto de estudio desde el siglo XIX. En 1858 William Guy publicó una investigación sobre las fluctuaciones promedio observadas en las defunciones según causas específicas en el período 1840-1854 en Londres (Inglaterra), señalando a la bronquitis y al asma como las de mayor amplitud luego de las enfermedades epidémicas. Esta variabilidad fue atribuida a las bajas temperaturas registradas en los períodos invernales ^1^.

Inglaterra, Gales y otros países europeos vigilan sistemáticamente este fenómeno desde la segunda mitad del siglo pasado, confirmando que registran mayores tasas de mortalidad en invierno respecto del verano ^2^. Sobre fines de la década de 1980, se creó el grupo colaborativo Eurowinter Group con el propósito de analizar el efecto que produce la caída de 1°C por debajo de los 18°C de temperatura ^3^. Los investigadores demostraron un incremento lineal en la mortalidad por cada grado de descenso de la temperatura por debajo de los 18°C y evidenciaron que este incremento porcentual en la mortalidad por todas las causas resultaba significativamente mayor en territorios con climas más cálidos.

La operacionalización de esta observación empírica fue realizada a través de la construcción de un indicador denominado exceso de mortalidad invernal, e índice de exceso de mortalidad invernal ^4^. Esta medida compara la cantidad de muertes acaecidas en el periodo invernal con el promedio de los períodos no invernales, no siendo solo expresión de la temperatura, sino también de otras dimensiones que operan tanto a nivel individual como colectivo. Public Health England lo considera un indicador de mortalidad prematura y de evaluación de políticas sociosanitarias ^5^. 

En países templados del Norte Global, las tasas de mortalidad invernal son entre 10% y 30% superiores a las no-invernales, siendo las poblaciones más añosas las más afectadas. Este fenómeno no se experimenta con igual magnitud en latitudes extremas, mencionándoselo como “la paradoja invernal” ^6^. Las causas subyacentes serían complejas y multidimensionales existiendo condiciones de posibilidad para que elementos ambientales, socioeconómicos, biológicos, culturales, políticos y de accesibilidad a los servicios de salud determinen vulnerabilidades diferenciales frente a la exposición a la temporada invernal ^7^.

En lo que respecta al Hemisferio Sur, este fenómeno ha sido estudiado con menor exhaustividad con hallazgos similares a los mencionados. Nueva Zelanda lidera las publicaciones al respecto, con un abordaje similar al del Reino Unido y otros países europeos ^8^. El estudio neozelandés fue la referencia operativa para esta investigación en lo que respecta a la periodización de la temporada invernal, sin embargo, comparte similitudes económicas, culturales y políticas con países del Norte Global. La bibliografía producida en Latinoamérica, aborda tangencialmente el problema, a través de estudios multicéntricos basados en la relación temperatura-mortalidad ^9^ o en el análisis de la mortalidad estacional por influenza.

Esta investigación adopta el principio de Uemura que establece que las tasas mínimas de la experiencia histórica de mortalidad alcanzadas por una población expresarían su capacidad de prevenir cualquier exceso hasta ese punto. Si los recursos estuvieran distribuidos equitativamente, no deberían naturalizarse diferenciales que son reducibles y reproducen inequidades ^10^. En función de sostener una vigilancia epistemológica respecto de la perspectiva desde donde abordamos esta investigación, explicitamos que, tal como enunciara Nancy Krieger ^11^, medidas tales como la mortalidad son expresiones biológicas de desigualdades sociales. Estas se manifiestan desde el nacimiento hasta la muerte mas no como innatas ni contingentes, sino más bien como impuestas y determinadas, operando tanto a nivel individual como colectivo ^12^.

El cambio climático introdujo nuevos debates alrededor del tema ^13^. El informe de *Lancet Countdown: Health and Climate Change in Latin America* demostró el calentamiento en todos los países de la región ^14^. Durante 2022, la población latinoamericana estuvo expuesta a temperaturas, en promedio, 0,38°C superiores que en el período 1986-2005. Solo Brasil, Chile y Uruguay cuentan con Planes Nacionales de Adaptación de la Salud (al cambio climático) ^14^. Dado que el índice de exceso de mortalidad invernal es un indicador resumen multidimensional, incluyendo dominios tales como salud, condiciones habitacionales, comportamiento, políticas energéticas y distribución del ingreso, resulta controversial cómo podría incidir el calentamiento global sobre la mortalidad invernal ^15^ ^,^ ^16^ ^,^ ^17^.

Hay cambios ya evidentes, como la epidemia de dengue que azotó a Argentina en la temporada 2023-2024 ^18^, producto del cambio climático y sus complejas interacciones con dimensiones eco-biológicas, sociales, políticas, económicas, en un contexto de políticas de ajuste ^19^. El escenario regional (a excepción de Brasil, Bolivia y México como más representativos) es el de nuevos populismos de derecha que acceden al gobierno democráticamente cooptando la insatisfacción del pueblo producto de demandas desatendidas por gobiernos socialdemócratas ^20^.

Frente a este contexto, es nuestro objetivo evidenciar la ocurrencia de exceso de mortalidad invernal en Argentina entre 1997-2017, describir su magnitud, tendencia y distribución para discutirlo desde una perspectiva crítica como objeto de conocimiento para la salud colectiva ^21^. 

## Metodología

Se realizó un estudio de series temporales, en el que se analizó el exceso de mortalidad invernal en Argentina entre 1997 y 2017 para el total país, regiones y provincias según sexo y grupos de edad. El criterio de agrupamiento de las regiones fue el utilizado por la Dirección de Estadísticas e Información de Salud del Ministerio de Salud de la Nación (DEIS) ^22^. Se exploró a nivel de estos agregados la presencia de diferenciales adoptando un diseño simultáneamente de agregados espaciales y temporales.

(1) Región Centro: Ciudad Autónoma de Buenos Aires (CABA), Buenos Aires, Córdoba, Entre Ríos, Santa Fe;

(2) Región Cuyo: La Rioja, Mendoza, San Juan, San Luis;

(3) Región Noroeste (NOA): Catamarca, Jujuy, Salta, Santiago del Estero, Tucumán;

(4) Región Noreste (NEA): Corrientes, Chaco, Formosa, Misiones;

(5) Región Patagónica: Chubut, La Pampa, Neuquén, Río Negro, Santa Cruz, Tierra del Fuego, Antártida e Islas del Atlántico Sur (TDF).

Para el análisis de series temporales, cada año del período 1997-2017 fue tomado como una unidad agregada completa ^23^. Para el análisis a nivel país (1997-2017), la población de estudio fue la estimada al 1 de julio de cada año calendario de la serie temporal ^24^.

Se incluyeron todas las defunciones registradas en el período comprendido entre el 1 de enero de 1997 y el 31 de enero de 2018 que constaban en las bases de datos de mortalidad de la DEIS las que no poseían datos identificatorios de los individuos (*Ley n. 17.622*) ^25^. Los grupos de edad fueron definidos acorde al estudio de Davie et al. ^8^: 0-4 años; 5-14 años; 15-29 años; 30-59 años; 60-79 años; 80 años y más según los años cumplidos al momento en que ocurrió la defunción. El sexo fue el registrado por la DEIS. El recorte temporal abarcó el período 1997-2017, dado que Argentina adoptó la utilización de la CIE-10 para la codificación de las causas de muerte el 1 de enero de 1997. 

La estacionalidad en la mortalidad fue analizada mediante el abordaje clásico de descomposición en componentes no observados según el método *Seasonal-trend decomposition procedure based on loess* (STL) ^26^. Este se aplicó a las tasas brutas y ajustadas (por edad) de la mortalidad mensual (según el método directo, utilizando la nueva población estándar de la Organización Mundial de la Salud 2000-2025) ^27^ ^,^ ^28^.

Para definir el período invernal de cada año (*t*), se aplicó la periodización utilizada en Nueva Zelanda ^8^. El período invernal fue definido como el cuatrimestre comprendido entre el 1 de junio y el 30 de septiembre de cada año calendario. El período no-invernal fue integrado por dos cuatrimestres, el primero correspondiente al verano y otoño precedentes (febrero-mayo) seguido por el cuatrimestre primavera/verano (octubre-enero) subsiguientes al período invernal de cada año. Es decir, que para cada año analizado *t*, el segundo cuatrimestre no invernal incluyó al mes de enero de *t +1*. 

A partir de la categorización de las muertes en invernales y no invernales, se calculó el exceso de muertes invernales y su índice según el método de la Office for National Statistics (ONS) ^4^ para los agrupamientos enunciados. El exceso de mortalidad invernal fue el resultado de la sustracción del promedio de las muertes no invernales al total de las defunciones invernales. El índice del exceso de muertes invernales (IEMI) fue la razón entre el exceso de mortalidad invernal (EMI) y el promedio de muertes no invernales, expresado como porcentaje, lo que permitió realizar comparaciones. Su intervalo de confianza fue estimado según el método de la ONS:

$$IC95\%\;IEMI=IEMI\pm 1,96\times \left(\frac{IEMI}{\sqrt{EMI}}\right)$$

Se estimaron medidas de tendencia central, dispersión y asociación acorde a la distribución del índice del exceso de muertes invernales, para total país, según edad, sexo, regiones y provincias. 

Si bien la ONS no aplica ajuste por edad del índice del exceso de muertes invernales, Public Health England reconoció que el índice sería en parte dependiente de la proporción de adultos mayores de la población ^5^. En concordancia con Fowler et al. ^29^, se realizó el ajuste por edad del índice del exceso de muertes invernales por el método directo ^27^. Se utilizó la prueba de suma de rangos con signo de Wilcoxon para muestras independientes para las comparaciones. Fueron consideradas estadísticamente significativas diferencias con valores p < 0,05. Los intervalos de confianza del 95% (IC95%) para las medianas fueron calculados mediante muestreo por reemplazo, obteniéndose nuevas medianas ordenadas. El rango entre los percentilos 2,5 y 97,5 compuso el IC95%.

Se exploró la relación entre la latitud y el índice del exceso de muertes invernales mediante regresión polinómica según la propuesta de Jones ^30^. La mediana de período del índice del exceso de muertes invernales de cada provincia fue tomada como variable dependiente y las variables independientes fueron la latitud y la longitud correspondientes al centroide geográfico de cada una. El modelo se seleccionó considerando los coeficientes de regresión, de determinación, el valor de p de los regresores, comportamiento de residuos y el criterio de información de Akaike (AIC, por su sigla en inglés).

Se compuso un mapa temático de Argentina (unicontinental para su mejor visualización) donde el color de relleno de cada provincia representó el quintil de la mediana del índice del exceso de muertes invernales del período. Si bien no contamos con las defunciones registradas en las Islas Malvinas por ser territorio ocupado por el Reino Unido de Gran Bretaña, le asignamos el quintil de la provincia de TDF, a la que pertenecen. La proyección cartográfica empleada fue la WGS 84 (World Geodetic System 1984), identificada por su código EPSG:4326.

Para el procesamiento y análisis estadístico se utilizó el software R versión 4.3.3 (http://www.r-project.org). El mapa fue creado con QGIS 3.22.7 (https://qgis.org/en/site/).

## Resultados

**Figura 1:**
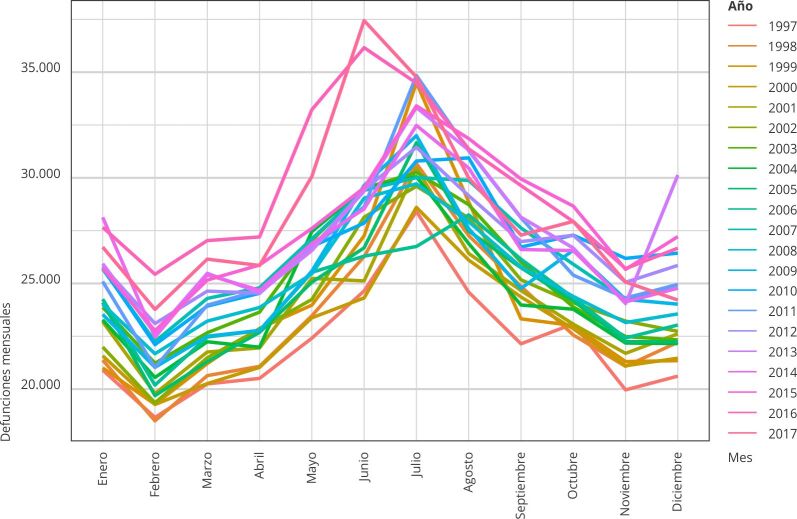
Análisis estacional de las defunciones mensuales en ambos sexos. Argentina, 1997-2017.

Se analizaron 6.422.932 defunciones, comprobándose, a nivel país, un exceso en las defunciones invernales respecto de las esperadas en períodos no invernales, del orden de las 407.950 muertes para la sumatoria de los 21 años que compusieron la serie. Se constató la presencia de un patrón estacional anual en la distribución de las defunciones, de magnitud mayor entre los meses de mayo y septiembre de cada año (Figura 1).

Fuente: elaboración propia en base a datos de la Dirección de Estadísticas e Información en Salud, Ministerio de Salud^22^

**Figura 2:**
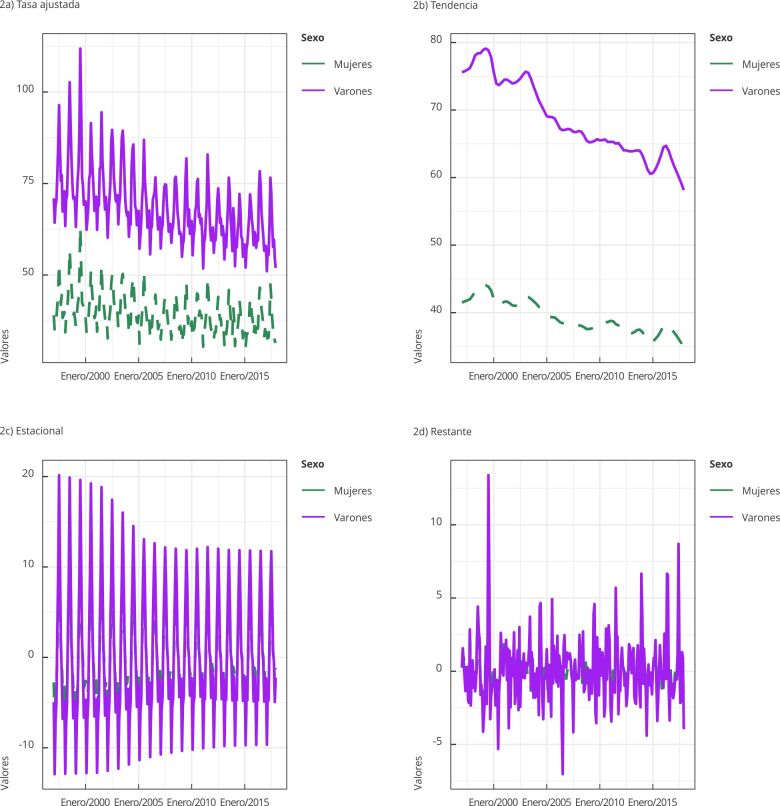
Descomposición de las tasas de mortalidad mensual ajustadas por edad (por 1.000 habitantes) en mujeres y varones. Argentina, 1997-2017.

La descomposición de la serie temporal de las tasas de mortalidad mensuales ajustadas por edad, exhibió un patrón (en mujeres y varones) de mayor magnitud en el período invernal (Figura 2). La fuerza de la componente estacional fue de 0,9 en ambos sexos, triplicando a la de la tendencia (0,3). El modelo aditivo de descomposición resultó adecuado ya que las oscilaciones en torno a la tendencia mantuvieron una amplitud similar a lo largo del tiempo. Se constató la presencia de sobremortalidad masculina en tasas brutas y ajustadas por edad. 

La componente estacional en mujeres presentó menor variabilidad en el tiempo. Tanto varones como mujeres presentaron el pico (*seasonal peak year*) de la componente estacional en el mes de julio, y su valle (*seasonal through year*) en febrero. Ambas series exhibieron autocorrelación significativa en sus tasas mensuales (coeficiente de Hurst = 0,9 en ambos sexos), indicativo de memoria de largo plazo. 

Se constató presencia de exceso de muertes invernales a lo largo de toda la serie temporal a nivel país y en ambos sexos. Esto representó, en promedio, un exceso de mortalidad invernal del orden del 20,4 % (IC95%: 18,6; 22,2), con un mínimo de 15% (IC95%: 14,7; 15,2) en 2010 y un máximo de 30,5% (IC95%: 30,2; 30,9) en 1999.

Las mujeres presentaron 212.370 defunciones invernales en exceso, mientras que los varones 194.768. El 92,3% del exceso se dio en mayores de 60 años, expresado predominantemente entre las mujeres en las mayores de 80 años (63%), mientras que en los varones en el grupo de 60-79 años (49,7%).

Las medianas del índice de exceso de mortalidad invernal estimadas para los datos agregados (sin ajuste) fueron de 21,8% en mujeres (IC95%: 20,5; 24,9) y de 18,2% en varones (IC95%: 16,2; 19,5). La prueba de Wilcoxon mostró una diferencia significativa entre las medianas de ambos grupos (valor de p < 0,001). La mediana del índice de exceso de mortalidad invernal ajustado fue también significativamente mayor en mujeres (19,5%) que en varones (18,3%) (p < 0,001; rbp = 0,92, IC95%: 0,81; 0,97). El coeficiente de correlación biserial de rangos (rbp) de 0,92 indicó una relación fuerte y positiva entre el sexo y el índice de exceso de mortalidad invernal, con valores de mayor magnitud en mujeres.

**Figura 3:**
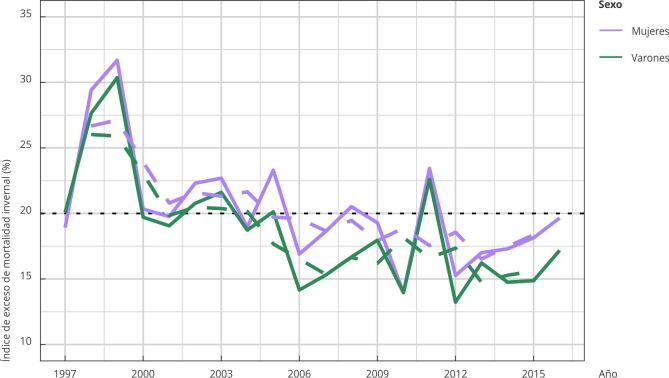
Índice de exceso de muertes invernales ajustado (%) y tendencia según sexo. Argentina, 1997-2017.

La serie temporal del índice de exceso de mortalidad invernal a nivel país se caracterizó por la ausencia de autocorrelación, presencia de exceso de mortalidad invernal en la totalidad del período, y mayor magnitud en mujeres (Figura 3). Se detectó una correlación positiva significativa entre los valores anuales del índice de exceso de mortalidad invernal entre mujeres y varones (coeficiente de correlación de Spearman (rs) = 0,92; IC95%:0,73; 0,98) para los valores ajustados. El índice de exceso de mortalidad invernal y la unidad temporal (año), presentaron correlación negativa más fuerte en varones que en mujeres.

Fuente: elaboración propia en base a datos de la Dirección de Estadísticas e Información de Salud, Ministerio de Salud ^22^. Nota: medias móviles de orden 3 para mujeres y varones (línea discontinua) y promedio de la serie temporal (línea punteada).

**Figura 4:**
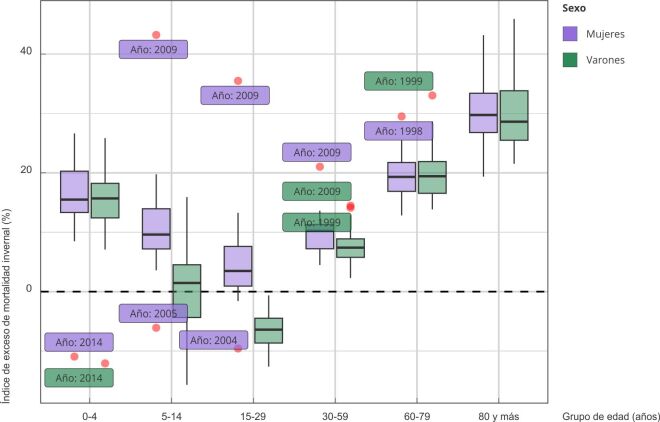
Distribución del índice de exceso de muertes invernales según sexo y grupos de edad. Argentina, 1997-2017.

La distribución del índice de exceso de mortalidad invernal a través de los seis grupos etarios presentó morfología en “V”, siendo mayor en menores de 5 años y desde los 60 años en adelante, con un nadir entre los 15 y 29 años (Figura 4). Las mujeres presentaron un IEMI significativamente mayor que los varones en los grupos de 5-14 años: 9,6% (IC95%: 7,2; 2,4) vs. 1,5%, (IC95%: -4,3; 2,9); p = 0,037 y 15-29 años: 3,5% (IC95%: 0,9; 5,9) vs. -6,4%, (IC95%: -8,6; -5,4); p < 0,001) alcanzando valores extremos en el año 2009. El grupo de 30-59 presentó solapamiento de los IC95% entre mujeres (10,2%; IC95%: 7,2; 10,6) y varones (7,4%; IC95%: 5,8; 7,9). No se encontraron diferencias significativas entre sexos en los grupos de mayor exceso de mortalidad invernal: 0-4, 60-79 y mayores de 80 años (datos no mostrados en la figura).

Fuente: elaboración propia en base a datos de la Dirección de Estadísticas e Información de Salud, Ministerio de Salud ^22^.

La comparación entre grupos de edad para cada sexo exhibió que las mujeres no presentaron diferencias significativas en la distribución del índice de exceso de mortalidad invernal entre los grupos de 0-4 años y 60-79 años. El grupo de mayores de 80 años presentó un índice de exceso de mortalidad invernal significativamente mayor a todos los grupos en ambos sexos. Entre los varones, el grupo de 0-4 años tuvo diferencias marginales con el de 60-79 años. A partir de los 30 años, los valores del índice de exceso de mortalidad invernal aumentaron significativamente con la edad. El grupo de 15-29 no presentó exceso de mortalidad invernal.

El modelo de regresión polinómica de orden 3 fue el seleccionado para modelizar la distribución del índice de exceso de mortalidad invernal en las provincias argentinas, mediante la ecuación:

$$y=17,0968-11,2611\times latitud-15,8385\times {latitud}^{2}+11,6479\times {latitud}^{3}$$

Los coeficientes de los términos polinómicos fueron significativos. El coeficiente de determinación (R^2^) fue = 0,78 (R^2^ ajustado = 0,75), con un error estándar residual de 2,66, y p < 0,0001. La prueba de Kolmogorov-Smirnov evidenció normalidad de los residuos. El AIC fue 120,6 y el factor de inflación de la varianza (VIF) de 1,81. El término de grado 1 mostró una disminución del índice de exceso de mortalidad invernal de 11,3 unidades por cada unidad de incremento en la latitud. Los términos de grado 2 y 3 capturaron curvaturas adicionales en la relación. El modelo mostró un desempeño similar al analizarlo por sexo.

**Figura 5:**
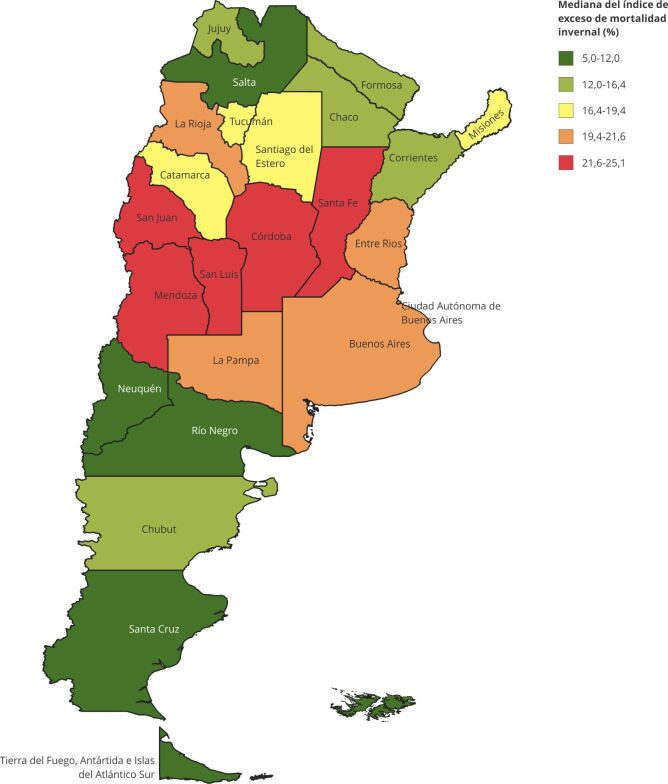
Mapa de la mediana del índice del exceso de muertes invernales de las Provincias Argentinas, 1997-2017, agrupadas por quintiles.

El mapa temático (Figura 5) exhibió que las provincias localizadas entre los 30° y los 37° de latitud Sur (regiones Cuyo y Centro) presentaron las mayores magnitudes del índice de exceso de mortalidad invernal, ubicándose entre el 4º y el 5º quintil. Las provincias patagónicas (latitudes más extremas), exhibieron menores diferenciales de mortalidad entre las temporadas invernales y no invernales. El resto, particularmente NEA y NOA presentaron un patrón heterogéneo en la distribución del índice de exceso de mortalidad invernal. 

Fuente: elaboración propia en base a Instituto Geográfico Nacional (https://www.ign.gob.ar/). Nota: se utilizó la versión unicontinental para mejor visualización.

## Discusión

En Argentina, entre 1997 y 2017 se produjo, anualmente, un exceso de mortalidad asociado a la temporada invernal. Las más de 400.000 defunciones en exceso, representan más que una cohorte de nacimientos. Esto nos condujo a preguntarnos ¿quiénes son los más vulnerables? Asumiendo que este exceso, es hasta cierto punto prevenible, y que además reproduce desigualdades, se transforma en objeto de análisis e intervención para la salud colectiva. Identificamos, además, una vacancia de conocimiento situada desde nuestro marco conceptual. 

La precisión y validez del índice de exceso de mortalidad invernal han sido cuestionadas, proponiéndose otras formas de medirlo, tales como los heating degree days (HDD) ^31^ ^,^ ^32^. Se plantearon sesgos de clasificación, de adelantamiento (*harvesting*), de exposición y también falacia ecológica. Recuperamos de la literatura posiciones diversas, particularmente sobre la relación temperatura - exceso de mortalidad invernal y calentamiento global ^9^ ^,^ ^33^. No obstante, captamos este exceso de mortalidad invernal en Argentina, que era nuestro propósito. Aplicamos un indicador inédito para visibilizar desigualdades en la distribución de la mortalidad.

Kephart et al. ^9^ analizaron el efecto en la mortalidad de las denominadas temperaturas no óptimas en Latinoamérica en su estudio sobre ciudades altamente urbanizadas. Evidenciaron que las muertes asociadas a la exposición a bajas temperaturas aportaron más a la mortalidad por todas las causas (5,09%; IC95%: 4,66; 5,42), que las asociadas a altas temperaturas (0,67%; IC95%: 0,58; 0,74) ^9^, en concordancia con el estudio de Zhao et al. ^33^, pero advierten sobre futuros escenarios asociados al calentamiento global. 

Guo et al. ^16^ expresan desde el Norte Global que más voces son necesarias desde el Sur Global. El Sur Global es un concepto geopolítico, una metáfora crítica ^34^, que incorpora la ecología de saberes para la construcción de conocimiento, un buen punto de anclaje para la salud colectiva. 

Nuestro estudio no incluyó la variable temperatura, sino la estacionalidad en la mortalidad y su exceso en el período invernal. Sostenemos que el análisis de la estacionalidad es más complejo, y que su asociación a la temperatura es solo parte necesaria, mas no suficiente, de un sistema de determinaciones. Coincidimos con Guo et al. ^16^ en que el invierno es un mediador más que un confusor a la hora de analizar la mortalidad invernal. Un estudio finlandés ^35^, analizó, desde el campo de la salud mental, la relación entre la estacionalidad de las enfermedades no transmisibles y el desorden afectivo estacional o Seasonal Affective Disorder (SAD). Señalan que el SAD tendría impacto sobre dimensiones biológicas, psicoafectivas, sociales, que inciden en el auto-cuidado, desencadenando complicaciones cardiovasculares y respiratorias, causas principales atribuidas al exceso de mortalidade invernal ^36^. 

La sobremortalidad masculina detectada en todos los grupos etarios se condice con la literatura, incluso aquella que tímidamente introduce la categoría género, desde la perspectiva performativa de lo que se espera de los roles y comportamientos de las personas cuyo sexo asignado al nacer es el masculino ^37^ ^,^ ^38^. McCreary et al. ^39^ introducen la categoría de auto-estigma para explicar la relación de los varones con el sistema de salud, adherencia a tratamientos y conductas asociadas a mortalidad prematura. En otro contexto socio-cultural, Trotta et al. ^38^ identifican diferenciales que denominan gender inequities en la esperanza de vida en la CABA, Argentina y caracterizan a los mecanismos que determinan esta brecha como elusivos. La Real Academia Española define a las acciones elusivas como aquellas que no tienen en cuenta alguna cuestión, de manera inadvertida o intencional, evitar es su sinónimo, aceptar su antónimo ^40^.

Desde la mirada decolonial de la salud colectiva, no “aceptamos” las inequidades, sino que intentamos transformarlas. La construcción de conocimiento situada desde el Sur Global es una forma de disputa epistémica. Así como la mortalidad general no se distribuye equitativamente en Argentina, tampoco lo hace el exceso de mortalidad invernal. Los diferenciales de mayor magnitud se presentaron entre 5 y 59 años, destacándose el año 2009, pandémico por influenza A H1N1pdm09. Un artículo de revisión publicado en *The Biology of Sex Diferencies*, también describe este diferencial en 2009 analizando sexo, género y embarazo ^41^. 

Nuestra variable de análisis fue sexo, pero en línea con Trotta et al. ^38^ y McCreary et al. ^39^ interpretamos que detrás del diferencial, subyacen determinaciones que también nos fueron elusivas. En nuestra investigación, el grupo entre 15-29 años, presentó el mayor diferencial entre mujeres y varones. Los varones de este grupo no presentaron exceso de mortalidad invernal, sugerente de otro patrón, tal vez vinculado al planteamiento de Trotta et al. acerca de las muertes violentas en hombres jóvenes. 

Las tareas de cuidado, remuneradas o no, en Argentina son ejercidas principalmente por mujeres reproduciendo las profundas desigualdades de género y clase producto de la división sexual del trabajo ^42^. Las mujeres, especialmente en contextos de menores recursos, llevan la mayor carga de estas responsabilidades. La escasa participación estatal (actualmente nula desde la asunción del Presidente Javier Milei en diciembre de 2023) y la preferencia cultural por el cuidado familiar agravan estas desigualdades. 

La feminización de la fuerza laboral en algunos sectores reproduce la crisis del sistema de cuidados. Esquivel & Pereyra ^43^ señalan que el sector del cuidado en Argentina está altamente feminizado, alcanzando un 98% de mujeres en el servicio doméstico y un 78% en los sectores de salud y educación. Nuestros resultados podrían sugerir exposiciones diferenciales a patógenos estacionales asociados a las tareas de cuidado. 

Respecto de la edad, los menores de 5 años y los mayores de 60 años, fueron la población más afectada por este exceso. Los valores exhibidos por los menores de 5 años, son superados a partir de los 60 años. Coincidimos con Jones ^30^ en el hallazgo que el exceso de mortalidad invernal no es privativo de los adultos mayores y que a partir de los 15 años se produce (en su estudio), un punto de inflexión descendente y en el nuestro, el nadir. El autor (utilizando grupos de edad quinquenales) no describe el exceso negativo que identificamos en varones de 15-29 años. Su estudio utilizó principalmente datos publicados por Naciones Unidas y Eurostat, por lo que más allá de la distinta conformación de los grupos etarios, los contextos son de difícil comparación.

Como mencionamos, tomamos al estudio neozelandés como referencia del Hemisferio Sur ^8^. Los autores analizaron varios predictores del exceso de mortalidad invernal, siendo la edad y el sexo femenino los más significativos. Coincidimos en identificar a los menores de 5 años y mayores de 60 como los más vulnerables a fallecer en temporada invernal, si bien la magnitud del exceso es inferior a la nuestra para todos los grupos etarios. Retomando la discusión de la comparabilidad, hemisferio sur es un concepto geográfico, mientras que Sur Global es político. 

Comprobamos que en Argentina se evidencia la paradoja invernal descripta por McKee ^44^ en 1989, con menor exceso en zonas de climas más fríos y latitudes más extremas, concepto revisitado por varios autores ^29^ ^,^ ^30^. Al igual que Jones, identificamos a las regiones cuyas latitudes rondan los 35°S (Centro y Cuyo), como las de mayor exceso. Nuestro modelo podría aportar al estudio chileno ^45^ (1984) que analizó la relación entre la mortalidad estacional, gradientes climáticos y latitudinales. Nuestros hallazgos en el NOA y NEA y los del autor en la región norte de Chile, podrían corresponder a los términos no lineales de nuestro modelo de regresión.

Nos interpela conocer la relación entre las determinaciones del exceso de mortalidad invernal y la mortalidad general. Ogbebor et al. ^36^ propone que el exceso de mortalidad invernal no varía en función de la mortalidad general de la población. Lo mismo sostiene el informe de la *National Bureau of Economic Research* ^31^, exhibiendo que la curva mensual del exceso de mortalidad invernal medido a través de los HDD, presenta en ambos sexos, mayor magnitud que la mortalidad general, alcanzando valores similares solo en los meses de julio y agosto. Coincidimos con esta tesis, y consideramos haber aportado a ella con nuestros resultados. El exceso de mortalidad invernal es una síntesis multidimensional de modos de vida colectivos y condiciones individuales, incluyendo categorías como pobreza energética, calidad constructiva de las viviendas, subsidios a tarifas energéticas, energías utilizadas. Estas determinan modos y conductas de afrontamiento de lo contingente de cada temporada invernal. 

Entre las limitaciones del estudio, si bien no fueron objetivos planteados, reconocemos que no presentamos las causas principales del exceso de mortalidad invernal, aunque nuestra línea de investigación sí lo exploró. En la intención de discutir el exceso de mortalidad invernal como expresión de desigualdades, nos focalizamos en responder las preguntas planteadas. Las implicancias de comunicar y debatir los resultados en este momento histórico, nos llevaron a asumirlas, tomando decisiones metodológicas para compartir este conocimiento, inédito en Argentina.

Como afirman Guo et al. ^16^ el impacto del cambio climático sobre Latinoamérica es evidente, siendo una de las regiones más urbanizadas del planeta, con mayores desigualdades y niveles de pobreza enraizados en nuestras sociedades. El escenario, en Argentina, no es promisorio frente al desmantelamiento de políticas sociales. Las condiciones de afrontamiento de la temporada invernal tanto a nivel individual como colectivo posiblemente empeoren, profundizando desigualdades existentes ^46^ ^,^ ^47^. 

El autodenominado gobierno anarco-capitalista de Argentina para el período 2023-2027 encarna una idea de libertad negativa ^48^. Este concepto se asemeja a la distopía de Orwell en 1984 “*freedom is slavery*” ^49^. En este sentido, las iniciativas en curso para desmantelar las políticas de transferencias monetarias y subsidios energéticos clausuran las posibilidades de emergencia de políticas redistributivas y justicia social apropiadas para mitigar los impactos del cambio climático y la reducción del exceso de mortlidad invernal, en nuestros contextos ^50^.

Este artículo aporta conocimiento relevante para la salud colectiva, ya que visibiliza, a través de un indicador *proxy*, desigualdades en la distribución de la mortalidad probablemente injustas y reducibles, sintetizando vulnerabilidades individuales y colectivas. La simpleza de su cálculo, con datos accesibles a nivel local, oportunos y situados en contexto, ofrece oportunidades de intervención desde distintos niveles de gestión y campos disciplinares junto a las comunidades.

La injusticia epistémica es parte de la injusticia social global. Disputar desde el Sur, el significado del significante “exceso de mortalidad invernal” constituye - en clave con las epistemologías del Sur - un acto de insurgencia epistémica ^51^. Nuestra tesis propone descolonizar su significado desde la salud colectiva para transformarlo en el territorio. Así, las comunas, municipios, cooperativas, organizaciones, se apropiarían críticamente de este conocimiento para problematizar los procesos y cadenas de determinación “elusivas” subyacentes. Este nuevo conocimiento construido colectivamente y en acto, tendría la potencialidad de ser plasmado en acciones comunitarias o en demandas políticas. De esta ecología de saberes podrían emerger nuevos discursos y prácticas que nos corran del nihilismo de época y protejan a los más vulnerables de morir precoz o injustamente cada temporada invernal.
